# Spatial Transcriptomics in Thyroid Cancer: A PRISMA-Style Systematic Review of Platforms, Architecture, and Clinical Applications

**DOI:** 10.3390/jcm15197636

**Published:** 2026-10-02

**Authors:** Harel C. Sims, Jason J. Sims, Monica M. Guirgus, Colby S. Grames, Alfred A. Simental, Salma Khan

**Affiliations:** 1 School of Medicine, Loma Linda University, Loma Linda, CA 92350, USA; hsims@students.llu.edu (H.C.S.); monicaguirgus@students.llu.edu (M.M.G.); cgrames@students.llu.edu (C.S.G.); 2 Department of Neurosurgery, University of Missouri School of Medicine, Columbia, MO 65212, USA; js7zp@health.missouri.edu; 3 Department of Otolaryngology-Head & Neck Surgery, Loma Linda University, Loma Linda, CA 92350, USA; asimenta@llu.edu; 4 Center for Health Disparities, Loma Linda University, Loma Linda, CA 92350, USA

**Keywords:** spatial transcriptomics, thyroid cancer, anaplastic thyroid carcinoma, tumor microenvironment, cancer-associated fibroblasts, immune microenvironment, metastasis, 10x Visium, GeoMx digital spatial profiling

## Abstract

**Background/Objectives:** Thyroid cancer spans indolent differentiated tumors to lethal poorly differentiated and anaplastic carcinomas, and histopathology and molecular testing do not capture the spatial organization of tumor, stromal, and immune compartments that underlies invasion, dedifferentiation, and treatment resistance. Spatial transcriptomics profiles gene expression in situ. We systematically reviewed its platforms, applications, and findings in thyroid cancer. **Methods:** PubMed/MEDLINE, Scopus, and Web of Science were searched, following PRISMA 2020 guidance and supplemented by citation chasing, for English-language primary studies applying spatially resolved transcriptomics to thyroid cancer tissue. Subtype, platform, complementary methods, and principal findings were extracted, and each study was appraised item by item with the design-matched Joanna Briggs Institute (JBI) checklist. **Results:** Of 104 records, 21 studies were included: 12 case series, four analytical cross-sectional studies, three single-case reports, and two reanalyses of one public dataset. Nineteen used capture-based whole-transcriptome platforms, predominantly 10x Visium, and two used GeoMx digital spatial profiling; papillary thyroid carcinoma predominated, and medullary carcinoma was not examined. Tumor-microenvironment remodeling accompanying dedifferentiation toward anaplastic carcinoma was replicated in five independent cohorts. Cancer-associated fibroblast activation was reported in three cohorts, although the SERPINE1-positive and POSTN-positive programs were each single-cohort; APOE-linked tumor states appeared in two cohorts with opposite directionality; and invasive-front extracellular-matrix dysregulation in follicular tumors was reported in two. Six studies reported functional perturbation and 12 protein-level validation; no biomarker had independent external validation. Methodological concern was low in four studies, moderate in seven, and high in 10: only one of 12 case series reported consecutive and complete inclusion, eight reported no outcomes for the profiled patients, and nine analyzed spots or cells rather than patients. **Conclusions:** Spatial transcriptomics has resolved reproducible microenvironmental remodeling during dedifferentiation, but its prognostic and mechanistic claims rest on small, selected cohorts without independent validation. Larger, consecutively enrolled, clinically annotated studies are needed.

## 1. Introduction

Endocrine malignancies are led in frequency by thyroid cancer, whose diagnosis rates have grown considerably across recent decades [1]. Thyroid neoplasms span a diverse histologic landscape under the 2022 World Health Organization framework. Tumors arising from follicular cells include papillary (PTC), follicular (FTC), oncocytic, poorly differentiated (PDTC), and anaplastic (ATC) carcinomas; these are joined by medullary thyroid carcinoma, a neoplasm traced instead to parafollicular C cells [2]. Indolence characterizes the natural history of most differentiated tumors, but not all: some return after treatment, seed distant organs, cease responding to radioiodine, or shed their differentiated phenotype to become PDTC or ATC. Of these, ATC ranks among the deadliest endocrine cancers, and patients frequently survive only months even after aggressive multimodal treatment [3]. Current risk stratification relies heavily on histopathologic features, anatomic staging, and selected molecular alterations; however, these approaches incompletely capture intratumoral heterogeneity, microenvironmental remodeling, and spatially organized tumor-stroma-immune interactions that contribute to invasion, immune escape, metastatic spread, and therapeutic resistance [4].

Traditional transcriptomic approaches have improved understanding of thyroid cancer biology but remain limited by loss of spatial context. Bulk RNA sequencing identifies tumor-level expression programs but returns one averaged profile per specimen, blending malignant, stromal, endothelial, and immune contributions. Single-cell RNA sequencing recovers per-cell resolution, but the dissociation step that makes it possible destroys the record of which cells were neighbors [5]. Spatial transcriptomics (ST) addresses this limitation differently: transcripts are assayed in situ, so every measurement carries the coordinates at which it was made on the histologic section [5,6,7]. The field was designated Nature Methods’ Method of the Year in 2020, reflecting its importance for linking molecular state to tissue architecture [6]. Tumors make this coordinate information consequential. A transcriptional program confined to the invasive boundary, to a lymphovascular interface, or to a dedifferentiated focus is diluted into the tissue average by any assay that discards position.

The value of positional information has been demonstrated most extensively outside the thyroid. Spatial profiling localized a tumor-specific keratinocyte population to a fibrovascular niche at the leading edge of cutaneous squamous cell carcinoma [8], resolved the co-localization of inflammatory fibroblasts with stress-response cancer cells in pancreatic ductal adenocarcinoma [9], defined stromal–immune “ecotypes” associated with outcome in breast cancer [10], and showed transcriptional alteration extending beyond the histologic tumor boundary in prostate cancer [11]. It has also distinguished tumor sub-regions and primary from metastatic lesions, coupling regional programs to hypoxia in glioblastoma [12] and revealing reprogramming toward immunosuppressive MRC1+CCL18+ macrophages in colorectal liver metastases [13]. The thyroid cancer studies reviewed here have begun to test the principles these studies established: spatially restricted tumor cell states, organized stromal and immune compartments, and spatially constrained cell–cell interactions.

Several classes of ST platforms are now in routine use, and each balances one capability at the expense of another. For example, subcellular localization is generally purchased with a preselected gene panel, while unbiased transcriptome-wide capture is purchased with spot-level averaging [14]. Capture-based platforms, derived from the original spatial transcriptomics approach described by Ståhl et al., profile RNA across spatially barcoded capture regions and are well suited for discovery-oriented whole-transcriptome studies [7]. Imaging-based platforms, including MERFISH, seqFISH-based methods, CosMx, and Xenium, provide single-cell or subcellular localization of targeted transcripts and are particularly useful for resolving cell states and cell–cell interactions at tumor boundaries [15,16,17]. Region-based platforms, such as GeoMx digital spatial profiling, quantify RNA or protein targets within pathologist-defined regions of interest and are compatible with formalin-fixed paraffin-embedded tissue, making them attractive for archival clinical specimens [18]. These complementary approaches are summarized in Table 1 and provide a framework for interpreting the strengths and limitations of the thyroid cancer ST literature.

Interest in applying ST to thyroid cancer has grown rapidly. Recent studies have used ST to characterize tumor-microenvironment remodeling across thyroid cancer differentiation states, fibroblast programs at invasive fronts, immune-evasion niches, spatial patterns of dedifferentiation, lymph node metastasis, and candidate biomarkers in ATC [19,20,21,22,24,25,26,27,29]. For example, ST studies have identified SERPINE1-positive [21] and POSTN-positive [22] cancer-associated fibroblast programs associated with aggressive behavior, APOE-linked malignant or immunosuppressive tumor states [21,26,29], and immune-suppressive, angiogenic, and extracellular-matrix remodeling during dedifferentiation toward ATC [20,24,25]. Narrative reviews have begun to summarize spatial and single-cell approaches in thyroid oncology [5]; however, a systematic, PRISMA-guided synthesis of the most recent advancements in the primary ST literature of thyroid cancer remains needed. Specifically, the field lacks a consolidated review that catalogs the platforms used, thyroid cancer subtypes studied, sample types analyzed, and biological findings reported by more than one independent investigation. A systematic review was therefore conducted to map the current ST platform landscape in thyroid cancer and synthesize the major applications, discoveries, limitations, and future directions of spatially resolved transcriptomics across thyroid cancer subtypes.

## 2. Materials and Methods

This systematic review was conducted and reported in accordance with the Preferred Reporting Items for Systematic Reviews and Meta-Analyses (PRISMA) 2020 statement [40]; the completed checklist is provided in Supplementary Table S4. The review was not prospectively registered. However, the eligibility criteria, search strategy, screening procedure, and extraction variables described here were documented in writing before screening began and were applied throughout without amendment, and no changes to eligibility were made after records had been screened. Because the objective was to map the available spatial-transcriptomics literature in thyroid cancer rather than estimate a pooled treatment effect, the review was designed as a qualitative evidence synthesis.

A systematic literature search was performed in PubMed/MEDLINE, Scopus (Elsevier, Amsterdam, The Netherlands), and Web of Science Core Collection (Clarivate, Philadelphia, PA, USA) with no lower date restriction; all three databases were last searched on 10 July 2026. Searches were limited to English-language records. Embase was not searched because of institutional access constraints; to mitigate the risk of missed studies, the database searches were supplemented by backward citation chasing and forward citation chasing (screening of records citing each included study, identified through the citing-article functions of Scopus and Web of Science). Records identified through citation chasing were screened against the same eligibility criteria as database records. Boolean search strategies combined terms related to spatial transcriptomics with terms related to thyroid cancer. Exact search criteria for each individual database are included in Supplementary Table S1 for reference.

Studies were eligible for inclusion if they met all of the following criteria: primary research; application of a spatially resolved transcriptomic method to thyroid cancer tissue or a thyroid cancer dataset; reporting of spatial gene-expression findings; and inclusion of thyroid-cancer-specific results. Studies using human thyroid cancer tissue were prioritized. Reanalysis of publicly available thyroid cancer spatial-transcriptomic datasets was included when they generated thyroid-cancer-specific spatial findings.

Studies were excluded if they were reviews, editorials, commentaries, conference abstracts without full-text data, non-thyroid studies, pan-cancer studies without thyroid-cancer-specific spatial-transcriptomic analysis, benign thyroid disease studies without cancer tissue, spatial-proteomics-only studies without a transcriptomic readout, technical methods papers without thyroid cancer application, studies without retrievable full text, or non-human model studies that did not include human thyroid cancer tissue.

Records were de-duplicated first by DOI and then by title. Search results from all databases were imported into EndNote (Clarivate, Philadelphia, PA, USA) for reference management, and de-duplication was performed in EndNote using its duplicate-detection function. Unique records were screened by title and abstract against the eligibility criteria by two reviewers (H.C.S. and J.J.S.) working independently, with disagreements resolved by consensus. Reports that appeared potentially eligible were retrieved for full-text review, and full-text articles were then independently assessed for final inclusion by the same two reviewers (H.C.S. and J.J.S.), with disagreements resolved by discussion. No automation tools were used during screening or selection. Dedicated screening platforms were not used because the searches yielded a small number of records (104 records; 47 unique after de-duplication in EndNote), which allowed title/abstract and full-text screening to be completed manually by the two reviewers within the EndNote library and cross-checked against a single shared record log.

Data were extracted into a standardized, pre-piloted extraction form by one reviewer (H.C.S.) and independently verified by a second reviewer (M.M.G.), with disagreements resolved by consensus, and study investigators were not contacted for additional data; when a variable was not reported or could not be determined from the published article or its Supplementary Materials, it was recorded as not reported (NR) in the extraction form and in Table 1, and no values were imputed or estimated. For each included study, extracted variables included first author, year of publication, journal, thyroid cancer subtype, cohort or sample size, tissue source, sample preparation method, spatial-transcriptomics platform, complementary methods, analytic approach, and principal spatial findings. Thyroid cancer subtypes were recorded as reported by the original studies. Spatial platforms were categorized as capture-based whole-transcriptome methods, region-based digital spatial profiling methods, imaging-based targeted transcriptomic methods, or integrated spatial multi-omic approaches.

Sample size was recorded hierarchically. The number of unique patients was the primary unit and is reported in Table 1 together with the number of tissue sections, capture areas, or regions of interest profiled by spatial transcriptomics. Spatial spots, regions of interest, and computationally segmented cells were treated as measurements within a section, not as independent samples. When several sections or samples were derived from the same patient, they were counted once at the patient level. A finding was regarded as replicated within a study only if it was observed across different patients, and findings resting on repeated sampling of a single tumor were treated as single-case evidence in the synthesis. For the two reanalyses of publicly available data, the accession numbers of the source datasets were cross-referenced against the included primary studies. When a reanalysis used data generated by an included primary study, the reanalysis and its source study were counted as a single line of evidence rather than an independent replication.

Complementary methods were also extracted when reported, including single-cell RNA sequencing, bulk RNA sequencing, whole-exome sequencing, immunohistochemistry, multiplex immunofluorescence, spatial proteomics, metabolomics, or computational deconvolution. Key findings were grouped by biological theme, including tumor-microenvironment remodeling, dedifferentiation, cancer-associated fibroblast programs, immune microenvironment organization, cell–cell communication, invasion, lymph node metastasis, distant metastasis, molecular classification, and candidate biomarker discovery.

Because no validated risk-of-bias instrument exists specifically for descriptive spatial-omics studies, included studies were appraised using the Joanna Briggs Institute (JBI) critical-appraisal checklist most closely matching each study design [41,42]. Single-case spatial-transcriptomics studies were assessed using the eight-item JBI checklist for case reports; descriptive multi-sample spatial-transcriptomics studies were assessed using the ten-item JBI checklist for case series; and comparative or outcome-oriented cohort studies were assessed using the revised eight-item JBI tool for the assessment of risk of bias in analytical cross-sectional studies [43]. The case-report and case-series checklists are the current JBI versions [42]; item numbering throughout follows the published tools. Design was assigned from the structure of each study’s spatial-transcriptomic component; Loberg et al. [22], whose spatial component is a descriptive set of selected tissue blocks although its bulk-cohort analyses are outcome-oriented, was therefore appraised as a case series. The two reanalyses of publicly available datasets [26,30], which did not fit any existing clinical JBI checklist, were appraised using a seven-item framework [44,45,46,47,48,49]. The appraisal instrument applied to each included study, and the rating of every checklist item for every study, are reported in Supplementary Table S2. This framework assessed data provenance, original-platform quality control, analytic pipeline description, batch-effect handling, independent or functional validation, code/data availability, and parameter transparency. These seven items were prespecified before appraisal and were adapted from published standards for sharing and reproducing spatial-transcriptomic data [44], community best-practice guidelines for single-cell and spatial analysis [45,49], guidance on batch effects in high-throughput data [46], the FAIR principles for data and code availability [47], and recommendations for reproducible computational research [48]. The framework is a structured reporting checklist and has not been formally validated.

Methodological appraisal was performed by one reviewer (H.C.S.) and independently checked by a second reviewer (C.S.G.), with disagreements resolved by consensus. A first-pass, item-level draft of the ratings was produced and annotated with the supporting passage and page of each article from the full text and Supplementary Files of every included study; both reviewers then checked each draft rating against the source article, and the consensus ratings were reported. Each appraisal item was rated as met, unclear/not reported, not met, or not applicable. Items that ask whether an element was reported (for example, inclusion criteria, participant demographics, clinical information, outcomes, or the presenting site) were rated not met when the element was absent from the article and its Supplementary Files and unclear when it was reported only in part; items that ask how the study was conducted (for example, consecutive or complete inclusion, reliability of measurement, or appropriateness of statistical analysis) were rated unclear when the procedure was not described and not met when it was described and judged inadequate. All checklist items were treated as applicable. Each study was appraised as published; where the spatial-transcriptomic component was a subset of a larger study, the size of that subset is noted in Table 1 and Supplementary Table S2.

An overall level of methodological concern was derived from the proportion of applicable items met: low concern, ≥75%; moderate concern, 50–74%; and high concern, <50%. The 75% and 50% cut points were prespecified. JBI does not prescribe thresholds for converting checklist responses into an overall rating and leaves this to the discretion of the review team [50]; the cut points used here follow the convention applied in prior reviews to summarize JBI checklist results into low, moderate, and high categories [51]; they were applied uniformly across the design-matched checklists and the seven-item framework for consistency and were not derived empirically. In addition to this checklist-completeness rating, each study was graded for risk of bias at the domain level. Checklist items were grouped into the bias domains they address: for case series, selection (items 1, 4, and 5), measurement of the condition (items 2 and 3), completeness of participant reporting (items 6, 7, and 9), outcome ascertainment (item 8), and analysis (item 10); for analytical cross-sectional studies, the categories and bias domains of the revised JBI tool [43]: selection (items 1 and 2), classification of the exposure (item 3), measurement of the outcome (item 4), confounding (items 5 and 6), statistical conclusion validity (item 7), and comprehensiveness of reporting (item 8); for case reports, patient description (items 1–3), diagnostic assessment (item 4), intervention and follow-up (items 5–7), and interpretation (item 8); and for the reanalyses, data provenance and quality control (framework items 1 and 2), analytic transparency (items 3, 4, and 7), and validation and availability (items 5 and 6). A domain was judged at low risk when every applicable item was met, moderate risk when at least half but not all items were met, and high risk when fewer than half were met, with unclear items counted as not met. The overall risk-of-bias judgment for a study was low when no domain was high and at most one was moderate, high when two or more domains were high or one domain was high and two or more were moderate, and moderate otherwise. Domain and overall judgments are reported in Supplementary Table S3. Because the checklists are adapted to descriptive spatial-omics studies and neither the cut points, the domain grouping, nor the seven-item framework has been validated against an external standard, these judgments are a structured and transparent grading of reporting completeness and of the structural sources of potential bias rather than validated measurements of study validity, and they were not used to weight or exclude studies.

Because the included studies were heterogeneous in spatial platform, tissue preparation, thyroid cancer subtype, sample size, analytic pipeline, and reported outcomes, meta-analysis was not appropriate. Findings were therefore synthesized qualitatively. Studies were first summarized descriptively by platform, sample type, thyroid cancer subtype, and use of complementary methods. Principal spatial findings were then organized into biological themes. A finding was classified as convergent only when the same cell population, gene program, or spatial relationship was reported in at least two studies drawn from independent patient cohorts; a reanalysis of a dataset generated by an included primary study was not counted as an independent cohort, and agreement between analytic approaches applied to the same tissue was not counted as replication. Single-case reports were treated as hypothesis-generating: they were not used on their own to establish any convergent finding. Convergence required at least one multi-patient study, and single-case origin is flagged in the evidence-strength summary (Table 2). For each finding in Table 2 we additionally recorded the number of independent patient cohorts, whether orthogonal validation (immunohistochemistry, immunofluorescence, protein-level spatial profiling, or flow cytometry) was reported, whether a functional perturbation experiment tested the proposed mechanism, and whether any proposed biomarker had been validated in an independent clinical cohort (Table 2). Associative spatial findings were thereby distinguished from experimentally supported mechanisms, and findings specific to a single subtype were distinguished from those supported across multiple thyroid cancer subtypes. Platform-specific strengths and limitations were considered when interpreting the evidence base. Because the synthesis was qualitative and did not include meta-analysis, formal statistical assessment of reporting bias across studies was not performed, and a formal grading of certainty of evidence was not applied; instead, confidence in the findings was contextualized qualitatively using the study-level methodological appraisal described above.

## 3. Results

### 3.1. Study Selection

The database search identified 104 total records, including 34 from PubMed/MEDLINE, 39 from Scopus, and 31 from Web of Science Core Collection. After removal of 57 duplicates, 47 unique records underwent title and abstract screening. Twenty-four records were excluded at this stage, most commonly because they were not thyroid-cancer-specific, were pan-cancer analyses without a thyroid-specific spatial-transcriptomic readout, were reviews or editorials, involved benign thyroid disease without cancer tissue, or lacked a transcriptomic spatial component.

**Figure 1:**
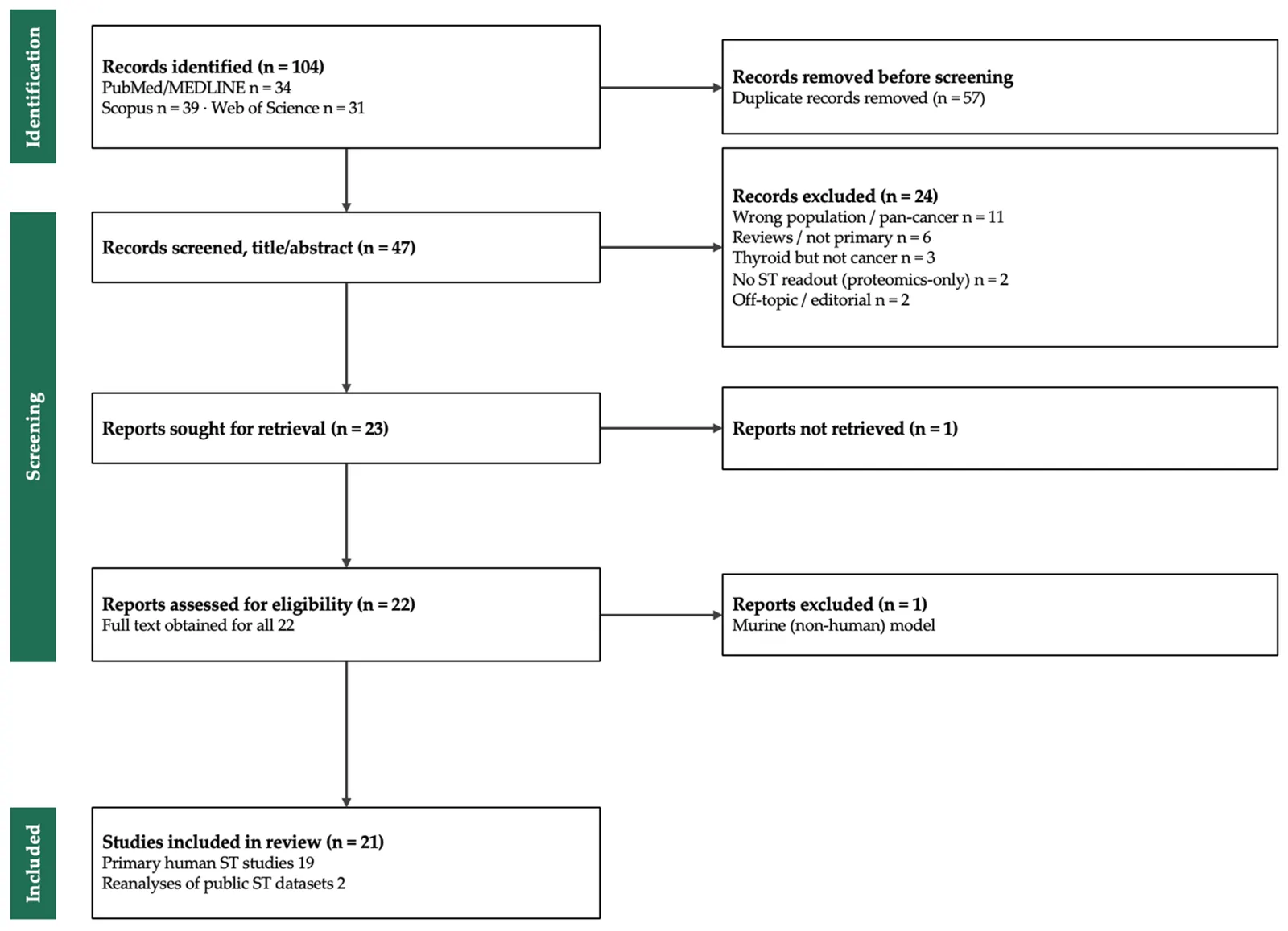
PRISMA 2020 flow diagram of study identification, screening, and inclusion.

Twenty-three reports were sought for full-text retrieval. One report could not be retrieved, leaving 22 full-text articles for eligibility assessment. One study using a genetically engineered murine model was excluded by consensus to restrict the synthesis to human disease-focused spatial-transcriptomic studies. Backward and forward citation chasing of the included studies identified no additional eligible reports beyond those already retrieved through the database searches. The study-selection process is summarized in the PRISMA flow diagram in Figure 1. Ultimately, 21 studies were included in the final qualitative synthesis, including 19 primary human spatial-transcriptomics studies and two reanalyses of publicly available thyroid cancer spatial-transcriptomics datasets. Cross-referencing of dataset accessions showed that both reanalyses [26,30] drew their spatial-transcriptomic data from GEO accession GSE250521, which was generated by the included primary study of Liao et al. [21]; findings shared between these reanalyses and Liao et al. were therefore counted as a single line of evidence. Two further overlaps were identified from the primary articles and their Supplementary Files: eight of the 15 anaplastic-carcinoma Visium sections analyzed by Loberg et al. [22] were generated for and first reported by Xu et al. [23], and Wang et al. 2024 [27] and Zhang et al. 2025 [28], from the same institution and author group, describe the same patient cohort (matching per-patient clinicopathological data in their Supplementary Tables). Findings shared within each pair were likewise counted once. The characteristics, spatial-transcriptomics platforms, thyroid cancer subtypes, complementary methods, and principal findings of these studies are summarized in Table 1.

### 3.2. Spatial Transcriptomics Platform Landscape

As summarized in Table 3, the spatial-transcriptomics platforms relevant to thyroid cancer can be broadly grouped into capture-based whole-transcriptome methods, region-based digital spatial profiling methods, and imaging-based targeted transcriptomic methods. Capture-based whole-transcriptome sequencing, particularly 10x Visium and Visium CytAssist, was the dominant approach across the included literature; study-level platform assignments are detailed in Table 1 [19,20,21,22,23,24,25,26,27,28,29,30,31,32,33,34,35,36,37,38,39]. This reflects the suitability of unbiased whole-transcriptome capture for discovery in surgical specimens, at the cost of 55 µm spot-level rather than single-cell resolution (Table 3).

The availability of FFPE-compatible workflows also makes Visium CytAssist particularly attractive for thyroid pathology, where archival tumor blocks are commonly available and histologic context is central to diagnosis [22,25,36,37].

Region-based GeoMx digital spatial profiling was used in studies requiring histology-guided profiling of defined regions of interest, particularly in FFPE-compatible biomarker-discovery settings [19,34]. GeoMx was useful for comparing tumor compartments or preselected histologic regions, but its region-level resolution limits direct single-cell interpretation. Higher-resolution capture-based platforms (Slide-seqV2, Stereo-seq, Seq-Scope) and imaging-based platforms (Xenium, CosMx, MERFISH, seqFISH+) were not used in any included study [15,16,17,52,53,54,55]; the latter would be the natural instruments for validating candidate cell states and ligand–receptor pairs at single-cell resolution.

### 3.3. Tumor Microenvironment Remodeling and Dedifferentiation

A dominant theme across the included studies was progressive remodeling of the tumor microenvironment during thyroid cancer progression and dedifferentiation. Several studies used ST, often integrated with single-cell RNA sequencing, to map the transition from differentiated thyroid carcinoma toward poorly differentiated thyroid carcinoma and ATC [20,21,24,25]. Liao et al. generated a spatially resolved transcriptomic landscape across para-tumor tissue, PTC, locally advanced PTC, and ATC, identifying three thyrocyte meta-clusters (TG+IYG+, HLA-DR+, and APOE+APOC1+) that marked successive stages of progression [21]. In the same study, SERPINE1+ fibroblasts increased with malignant progression and were associated with prognosis, supporting a role for fibroblast remodeling in aggressive thyroid cancer biology [21].

Ning et al. showed that ATC regions were enriched for immune suppression, angiogenesis, and extracellular-matrix remodeling and, by whole-exome sequencing and inferred copy-number analysis of the same specimens, that adjacent differentiated regions already carried thyroid-cancer-associated driver mutations and a tumor mutational burden approaching that of the anaplastic component, consistent with genomic priming before morphologic dedifferentiation [24]. Multiregion exome sequencing of coexisting components supports this interpretation: the anaplastic component shares truncal drivers (most often BRAF V600E or RAS) with the differentiated component but carries additional TP53 and TERT promoter alterations and can diverge early from the differentiated clone [56,57,58]. Ning et al. remains the only included study to have sequenced DNA from distinct tumor regions; Loberg et al. related the POSTN+ fibroblast program to BRAF V600E status only at the whole-tumor level [22].

The immune microenvironment of ATC was a consistent axis of this remodeling. From PTC and FTC to PDTC and ATC, Seok et al. found rising myeloid-cell and cancer-associated-fibroblast content with falling natural killer and endothelial cells, a myeloid-derived suppressor cell score inversely related to the thyroid differentiation score, and shifts in JAK-STAT and VEGF pathway activity [25]; Ning et al. found thymidine phosphorylase–positive (TYMP+) tumor-associated macrophages enriched in ATC regions and showed in vitro that loss of programmed cell death 4 (PDCD4) in tumor cells drives an eIF4A-dependent immunosuppressive program [24]; and Wang et al. described SFRP4-high tumor-specific myeloid cells accompanying dedifferentiation and poor prognosis in ATC and its metastases [39]. Metabolic reprogramming was a second axis, reported in three cohorts: glycolytic, lactate-secreting fibroblasts in poorly differentiated regions (ZFP57–PKM2 axis) that promote tumor-cell dedifferentiation, an effect reversed by resveratrol in vitro [20]; enhanced aerobic metabolism with suppressed translation programs in spatially defined PTC regions [31]; and dysregulated arginine–polyamine, glycolytic, and lipid pathways mapped by spatial metabolomics onto PTC tumorigenesis and lymph node metastasis [38]. Spatial metabolic measurements in ATC tissue have not been reported, so the ATC-specific evidence for metabolic reprogramming rests on the fibroblast glycolysis program alone [20].

Cancer-associated fibroblast programs recurred across cohorts: SERPINE1+ fibroblasts that increased with progression [21], POSTN+ myofibroblast-like CAFs abutting invasive tumor cells and linked to poor prognosis and lymph node metastasis [22], and glycolytic ZFP57–PKM2 CAFs [20], each reported in a single cohort (Table 3). Dedifferentiation therefore appears spatially coupled to fibroblast activation, extracellular-matrix remodeling, immune suppression, and metabolic reprogramming rather than being a malignant-cell-intrinsic process alone.

### 3.4. Cellular Heterogeneity, Cell States, and Molecular Classification

Several studies used ST to resolve spatial and clonal heterogeneity that is difficult to capture with bulk sequencing or dissociated single-cell approaches alone. Zheng et al. integrated single-cell RNA sequencing with spatially resolved transcriptomics to reconstruct the spatial evolution of PTC and improve molecular classification based on spatially organized malignant and microenvironmental features [31]. Yan et al. similarly used ST to identify prognosis-associated cellular heterogeneity within the PTC microenvironment and derived spatial RNA-clinical signature genes that mapped clinically relevant cell-type distributions across tissue sections [32].

Proteogenomic and multi-omic studies further demonstrated how ST can refine molecular classification in advanced thyroid cancer. Zhang et al. incorporated spatial transcriptomics into a proteogenomic analysis of advanced differentiated thyroid cancer and identified clinically relevant subtypes, including canonical, stromal, and immunogenic groups [33]. Xu et al. developed a Molecular Aggression and Prediction score that integrated immune-microenvironment features and improved outcome prediction in aggressive thyroid malignancy [23].

Beyond classification, ST contributed to prognostic biomarker development in three ways. It identified candidates by localizing prognosis-associated programs to defined compartments: relapse-free-survival signature genes and ligand–receptor pairs from a single tumor focus [32], and the SERPINE1+ and POSTN+ fibroblast programs from their distribution at progressing or invasive regions [21,22]. It characterized candidates by placing them in architectural context, as with the APOE/NCF1 immunosuppressive niche [26] and the lymph node metastasis signature and random-forest model of Jiang et al. [30], both derived from public spatial data. And it served as a validation layer, in independent spatial and single-cell cohorts (the proteogenomic DTC subtypes [33]), in bulk RNA-sequencing cohorts (the myCAF signature in five cohorts [22]; SERPINE1+ fibroblasts in an in-house cohort and TCGA [21]), and in protein-level assays (eight ATC markers on tissue microarrays [19]). None of these candidates has been validated in an external clinical cohort by independent investigators (Section 3.10; Table 2), so all remain at the discovery or internal-validation stage.

### 3.5. Immune Microenvironment and Cell–Cell Interactions

Spatial transcriptomics repeatedly revealed organized immune niches and tumor-immune cell communication patterns in thyroid cancer. Wang et al. showed that interactions between lysosome-associated membrane protein 3-positive (LAMP3+) dendritic cells and T-cell subpopulations promoted immune evasion in progressive PTC [27]. Shobab et al. used GeoMx digital spatial profiling and flow cytometry to identify sex-specific differences in the immune microenvironment of differentiated thyroid cancer, including higher dividing natural killer cells and T-cell immunoreceptor with immunoglobulin and immunoreceptor tyrosine-based inhibitory motif (ITIM) domains-positive (TIGIT+) CD8 T cells in male patients [34].

Several studies identified ligand–receptor axes that may mediate tumor-microenvironment crosstalk: the protein S (PROS1)–MER tyrosine kinase (MERTK) axis in papillary thyroid microcarcinoma [28], an APOE/neutrophil cytosolic factor 1 (NCF1)-associated immunosuppressive niche with prognostic significance derived from public data [26], and a C-X-C motif chemokine ligand 8 (CXCL8; interleukin-8)-positive monocyte–syndecan-1 (SDC1; CD138)-positive tumor-stem-cell network associated with distant metastasis [35]. In the last study, cell–cell communication inferred from single-cell data (CellChat) and mapped onto Visium sections by deconvolution (SPOTlight) placed CXCL8+ monocytes near SDC1+ tumor stem-like cells with JAK-STAT signaling downstream; both genes were higher in a high-risk, poor-prognosis transcriptomic subtype; and in monocyte–tumor stem cell co-culture and nude-mouse xenografts the axis increased sphere and colony formation, migration, subcutaneous growth, and lung metastasis of FTC-238-derived stem-like cells [35]. Because patient numbers were not stated, proximity was inferred rather than imaged, and the in vivo work used xenografts, its contribution to spontaneous distant metastasis in patients remains to be established (Table 2).

### 3.6. Invasion, Lymph Node Metastasis, and Distant Metastasis

Because capsular and vascular invasion define malignancy in follicular-patterned tumors, several studies examined the invasive front. In a single FTC, Condello et al. found that front-associated tumor clusters overexpressed extracellular-matrix-remodeling and epithelial–mesenchymal transition genes, that phylogenetic reconstruction placed these clones late in tumor evolution, and that two front-enriched genes, POSTN and DPYSL3, showed a core-to-periphery gradient by immunohistochemistry in an independent cohort of follicular tumors [36]. Suzuki et al. localized a CD74-high subpopulation to the invasive area of a minimally invasive FTC and confirmed higher peripheral than central CD74 immunoreactivity in 36 FTCs [37]. In PTC and ATC, SERPINE1+ fibroblasts accumulated at the leading edge with progression, and a SERPINE1–PLAUR fibroblast–macrophage interaction was inferred in ATC [21]; POSTN+ myofibroblastic CAFs directly abutted LAMB3+ partial-EMT invasive tumor cells, with inflammatory CAFs positioned distant from the tumor [22]. Across subtypes, the leading edge therefore pairs a matrix-remodeling fibroblast compartment with tumor cells that have acquired partial mesenchymal, invasion-associated gene expression.

Spatial approaches also provided insight into lymph node metastasis. Xiao et al. identified an APOE-negative tumor-cell subpopulation associated with pathological lymph node metastasis in advanced PTC and showed that APOE modulated proliferation and invasion [29]. Jiang et al. used public ST data integrated with single-cell and bulk transcriptomic datasets to develop a lymph node metastasis signature and machine-learning model for N1 versus N0 stratification [30]. Li et al. combined spatial metabolomics with Visium transcriptomics to map metabolic and transcriptional changes associated with PTC tumorigenesis and lymph node metastasis [38].

Distant metastasis was profiled spatially in one study. Wang et al. placed PTC, ATC, and ATC lymph node and gastric metastases in the same spatial dataset, identifying SFRP4-high tumor-specific myeloid cells associated with dedifferentiation and poor prognosis and localized near CD44+ tissue-resident memory T cells, with CD44+PKHD1L1+ tumor cells enriched in the metastases relative to the primary tumor [39].

### 3.7. ATC Biomarker Discovery

Six studies included ATC tissue [19,20,21,24,25,39]. Five profiled ATC alongside differentiated or poorly differentiated tumor along the dedifferentiation continuum [20,21,24,25,39], and Haq et al. compared ATC with PTC and other non-anaplastic follicular-cell-derived carcinomas by GeoMx digital spatial profiling of a single index case, identifying eight mRNA and protein markers of ATC progression (including COL7A1, LAMC2, SPHK1, and SRPX2) that were confirmed at the protein level on tissue microarrays [19]. Across these six studies the ATC compartment was consistently characterized by immune suppression, extracellular-matrix remodeling, angiogenesis, fibroblast activation, metabolic reprogramming, and myeloid-cell infiltration (Section 3.3; Table 2).

### 3.8. Summary of Included Studies

The 21 studies are summarized in Table 1. Single-cell RNA sequencing was the most frequent complementary method (11 of 21 studies, three of them using public single-cell data), followed by immunohistochemistry or immunofluorescence (12 of 21), functional assays (6 of 21), and, in one study each, whole-exome sequencing [24], proteogenomics [33], flow cytometry [34], and spatial metabolomics [38]. PTC was the most frequently studied subtype; FTC was profiled in three studies [25,36,37], PDTC in three [20,24,25], ATC in six [19,20,21,24,25,39], distant metastatic disease in two [35,39], and medullary thyroid carcinoma in none.

### 3.9. Critical Appraisal and Risk of Bias

The 19 primary studies were appraised with the JBI checklist matched to their design (12 case series, four analytical cross-sectional studies, three single-case reports) and the two reanalyses [26,30] with the seven-item framework. The rating of every item for every study is given in Supplementary Table S2 and the domain-level risk-of-bias judgments in Supplementary Table S3. Overall methodological concern was low in four studies (Xu et al. [23], Zhang et al. 2026 [33], Shobab et al. [34], and Ning et al. [24]), moderate in seven [19,21,26,28,30,31,39], and high in 10 [20,22,25,27,29,32,35,36,37,38]; when the same ratings were grouped into bias domains, the overall risk of bias was low in three [23,24,33], moderate in four [19,26,30,34], and high in 14. The pattern was the same across designs: the condition was almost always identified and measured reliably, whereas how patients were selected, who they were, what happened to them, and at what level the data were analyzed were frequently unreported.

Among the 12 case series, 11 identified the thyroid cancer subtype histopathologically and eight described how the diagnosis was confirmed, most often by independent review by two pathologists (items 2 and 3; not described in [22,29,32,35]). Selection was the weakest area: two studies stated clear inclusion criteria [24,31], three stated none [22,29,32], and seven defined their groups without describing how cases were chosen (item 1); only Ning et al. [24] described consecutive and complete inclusion (items 4 and 5), the other 11 being convenience samples of surgical specimens. Reporting of the profiled patients was uneven: demographics were absent in four [20,27,35,38] and limited to an adult/pediatric designation in one [22] (item 6); clinical information was partial in four [20,22,35,38] (item 7); and only four fully described the presenting site, with institution and recruitment period [20,24,35,39] (item 9). Outcomes or follow-up were reported in three [24,27,28], absent in eight [20,21,22,29,31,32,35,38], and confined to a single index patient in one [39] (item 8). Statistical analysis was appropriate in three [21,22,24], which compared groups at the level of patients or sections, and unclear in nine [20,27,28,29,31,32,35,38,39], which tested thousands of spots or cells from two to eight patients or did not name the test (item 10). Items met ranged from 1 to 10 of 10: Ning et al. was rated low concern, four studies moderate concern [21,28,31,39], and seven high concern [20,22,27,29,32,35,38].

The four analytical cross-sectional studies [23,25,33,34] all described their subjects and setting, used standard criteria for the condition, and measured outcomes validly (items 2, 4, and 8). Seok et al. [25] fell short on the remaining items: its 12 cases were selected without stated criteria, the exposure and confounders were handled unclearly, and comparisons were made at spot level (items 1, 3, 5, 6, and 7), so it was rated high concern. The other three were rated low concern with two shortcomings: strategies to address confounding were stated only by Xu et al. [23] (item 6), and the analysis of Shobab et al. [34] (region-level differential expression across nine patients without stated adjustment) was unclear (item 7). In all three, moreover, the spatial component was a small subset of the parent study (8 anaplastic-carcinoma sections of 312 samples [23]; 4 of 10 validation patients [33]; 9 of 27 patients [34]) whose selection was not described.

The three single-case studies [19,36,37] were appraised with the eight-item case-report checklist with all items applicable. All three provided take-away lessons (item 8), and Haq et al. [19] and Condello et al. [36] described the patient’s demographics and the diagnostic and molecular work-up (items 1 and 4), but only Haq et al. described the treatment (thyroidectomy with neck dissection, chemotherapy, and radiation) and the subsequent course (progression at 14 months and death; items 5 and 6). None presented the history as a timeline, fully described the condition at presentation, or addressed adverse events (items 2, 3, and 7), and Suzuki et al. [37] reported no individual data for the index patient. Haq et al. met 5 of 8 items (moderate concern); Condello et al. (3 of 8) and Suzuki et al. (1 of 8) were rated high concern.

Both public-data reanalyses [26,30] described their pipeline, batch-effect handling (Harmony in both, ComBat additionally in [26]), and parameter choices, and validated their signatures in independent bulk datasets (an external cohort in [26]; internal and external test sets and an in vitro knockdown in [30]) (items 3, 4, 5, and 7). Neither reported quality control of the reused spatial sections at the original platform level (item 2), cited the primary study that generated GSE250521 [21], or released code (unavailable in [26]; available on request in [30]; item 6), and the single-cell source stated by Su et al. [26] (GSE60542) is a bulk expression dataset, leaving its provenance unclear (item 1). Su et al. met 4 of 7 items and Jiang et al. [30] 5 of 7 (both moderate concern).

The domain-level grading (Supplementary Table S3) locates these deficiencies. Selection was rated high risk in 11 of 12 case series (all but Ning et al. [24]) and moderate in one cross-sectional study [25]; outcome ascertainment was high risk in nine case series [20,21,22,29,31,32,35,38,39] and analysis in nine [20,27,28,29,31,32,35,38,39]; and participant reporting was high risk in five [20,22,27,35,38]. Measurement of the condition, by contrast, was low risk in eight case series and in all four cross-sectional studies. Among the cross-sectional studies, confounding was the weakest domain (high risk in [25]; moderate in [33,34]); classification of the exposure was high risk in Seok et al. [25], and statistical conclusion validity in two [25,34]; the reanalyses were graded moderate overall, driven by data provenance and quality control (high in [26], moderate in [30]) and by code availability. No study was excluded on these grounds; the judgments were used to contextualize confidence in the findings synthesized in Table 2, in particular the prognostic associations reported by studies with high selection or reporting concerns.

### 3.10. Strength of Evidence for the Principal Findings

Table 2 summarizes the thyroid cancer subtypes, supporting studies, independent patient cohorts, and validation methods for the principal findings. Tumor-microenvironment remodeling accompanying dedifferentiation was the only finding replicated across more than two independent primary cohorts [20,21,24,25,39]. Cancer-associated fibroblast activation was observed in three cohorts, although individual marker programs—including SERPINE1+ [21], POSTN+ [22], and ZFP57–PKM2 [20]—were each reported in a single cohort. APOE-associated tumor states were identified in two independent cohorts [21,29], but with opposite directionality, while the APOE/NCF1 niche reported by Su et al. [26] was derived from the Liao et al. dataset and therefore does not represent independent replication. Organized immune-evasion niches were supported by one primary cohort [27] and two reanalyses [26,30] of another [21]. Invasive-front extracellular-matrix dysregulation in follicular tumors was reproduced in two independent cohorts [36,37].

Functional perturbation was performed in six studies [20,24,28,29,35,38], including experiments targeting the ZFP57–PKM2–lactate, PDCD4–eIF4A, APOE, CXCL8–SDC1, and PROS1–MERTK pathways. However, all functional validation was conducted by the same groups that generated the spatial findings, and none has been independently replicated. Orthogonal protein-level validation was reported in 12 studies [19,21,22,23,24,25,27,28,29,34,37,39]. No proposed biomarker, prognostic signature, or classifier has yet been externally validated in an independent clinical cohort; reported validation was limited primarily to internal cross-validation or public bulk transcriptomic datasets.

Across subtypes, the most consistent shared findings were progressive tumor-microenvironment remodeling and stromal, immune, angiogenic, extracellular-matrix, and fibroblast reprogramming along the differentiated-to-anaplastic continuum [20,21,22,24,25,39]. Other findings were largely subtype-specific: immune-evasion, metastatic, metabolic, and prognostic programs predominated in PTC [27,28,29,30,31,32,38]; invasive-front extracellular-matrix and CD74 changes were described in follicular-patterned tumors [36,37]; sex-specific immune differences were reported in differentiated thyroid cancer [34]; and digital spatial profiling markers were specific to ATC [19]. No spatial-transcriptomic studies of medullary thyroid carcinoma were identified.

## 4. Discussion

This systematic review identified 21 studies applying spatial transcriptomics to thyroid cancer, published between 2023 and 2026: 19 primary studies (12 case series, four analytical cross-sectional studies, three single-case reports) and two reanalyses of a single public dataset. Their distinctive contribution is positional: programs confined to invasive fronts, dedifferentiated foci, or immune niches were resolved that bulk sequencing averages away and dissociated single-cell methods cannot place.

Five independent cohorts [20,21,24,25,39] showed that dedifferentiation toward PDTC or ATC is accompanied by coordinated remodeling of the tumor microenvironment (fibroblast activation, extracellular-matrix remodeling, angiogenesis, immune suppression, and altered metabolism), the only finding replicated beyond two cohorts (Table 3). Fibroblast activation was seen in three of these cohorts, but the SERPINE1+ [21], POSTN+ [22], and ZFP57–PKM2 [20] programs were each described in one, so their proposal as prognostic markers or therapeutic targets rests on single-cohort evidence.

Four studies addressed immune organization: LAMP3+ dendritic cell–T cell interactions in progressive PTC [27], the APOE/NCF1 immunosuppressive niche derived from public data [26], SFRP4-high myeloid cells in ATC and its metastases [39], and CXCL8+ monocyte–SDC1+ tumor stem-cell signaling in metastatic disease [35]; two had protein-level confirmation [27,39] and one functional support [35]. Given the variable response of ATC to immune-checkpoint inhibition [3], spatial maps of inflamed, immune-excluded, and immune-suppressed regions could guide combination therapy, but none has yet been related to treatment response.

In follicular-patterned tumors, two single-case studies compared invasive fronts with tumor cores [36,37], each extended by immunohistochemistry (POSTN and DPYSL3 gradients in an independent follicular-tumor cohort [36]; CD74 in 36 FTCs [37]). In PTC, lymph node metastasis was associated with a functionally tested APOE-negative tumor-cell state [29], a computational signature derived from public data [30], and metabolic programs validated in zebrafish xenografts [38]. Distant metastases were profiled spatially in one study, which found SFRP4-high myeloid cells and CD44+PKHD1L1+ tumor cells enriched in ATC metastases [39].

Functional perturbation was reported in six studies [20,24,28,29,35,38] (ZFP57–PKM2 lactate signaling [20], PDCD4 loss–driven immunosuppression [24], APOE-dependent proliferation and invasion [29], metabolic programs of lymph node metastasis [38], and the CXCL8–SDC1 [35] and PROS1–MERTK [28] axes), but always within the cohort that generated the hypothesis; the remaining findings, including the dendritic cell–T cell and APOE–NCF1 interactions inferred from expression data, are associative. Because most studies examined PTC, the dendritic cell–T cell program, the PROS1–MERTK axis, and the lymph node metastasis signatures should not be extrapolated to follicular, poorly differentiated, or anaplastic disease without direct evidence.

Age is a further source of heterogeneity. Pediatric PTC is more often driven by RET and NTRK fusions than by BRAF V600E and carries a higher risk of nodal and pulmonary metastasis [59], and its immune and fibroblast composition differs from adult PTC at single-cell resolution [60]. Only Loberg et al. profiled pediatric tumors spatially (eight RET fusion–driven PTCs alongside adult PTC and ATC): tumor-adjacent POSTN+ myofibroblastic CAFs, tumor-distant inflammatory CAFs, and an abutting LAMB3+ partial-EMT invasive population were conserved across ages, whereas RET-driven pediatric tumors showed greater myCAF abundance and higher POSTN and FAP expression [22]. No study compared pediatric and adult immune composition at spatial resolution.

Technologically, the literature is concentrated: 19 of 21 studies used capture-based whole-transcriptome platforms, predominantly 10x Visium, two used GeoMx digital spatial profiling, and none used an imaging-based platform (Table 1 and Table 2). A 55 µm Visium spot may overlie several cells, so malignant, stromal, and immune signal arrive summed; the included studies mitigated this with single-cell annotation (11 studies), deconvolution, protein-level confirmation (12 studies), or external datasets. Imaging-based platforms that resolve single cells (Xenium, CosMx, MERFISH, seqFISH+) remain unused in thyroid cancer and are the natural next step for validating candidate cell states and ligand–receptor interactions [15,16,17,55].

The translational opportunities are concrete but unrealized: biomarker discovery anchored to tissue location, retrospective profiling of FFPE archives linked to outcome, stromal- and immune-informed classification, and target discovery in ATC. None of the candidates nominated so far (SERPINE1, POSTN, CD74, the MAP score, the proteogenomic DTC subtypes, or the APOE/NCF1 and lymph node metastasis signatures) has been validated in an external clinical cohort by independent investigators (Table 2).

Preoperative cytology frames the clinical need. The 2023 Bethesda System assigns implied malignancy risks of about 4% to Bethesda II and 22% to Bethesda III nodules [61], whereas surgical series report malignancy in 30–38% of resected Bethesda III nodules [62,63,64] and in 3–21% of cytologically benign nodules, higher for nodules ≥4 cm and in women [62,65]. Spatial transcriptomics cannot be applied to aspirates, but the spatially restricted programs it resolves, such as invasive-front extracellular-matrix and CD74 signatures in follicular-patterned tumors [36,37], nominate markers that, if validated in cytologic material, could refine risk stratification of Bethesda II and III nodules.

The limitations of the evidence base are quantifiable. Spatially profiled patients numbered 14 or fewer in 17 of the 19 primary studies, 26 tumors (28 sections) in one [22], and were not stated in one [35]; no study related a spatial finding to treatment response; FTC, PDTC, medullary carcinoma, and distant metastatic disease were each profiled in three or fewer studies; and platforms, tissue preservation, section selection, normalization, deconvolution, and cell-type annotation differed across studies, precluding meta-analysis. The review searched three databases in English only, without Embase, grey literature, trial registers, or conference proceedings, and was not prospectively registered; citation chasing identified no additional eligible reports, but language and publication bias cannot be excluded. The evidence-strength categories in Table 2 rely on what the original studies reported and may understate validation performed but not described.

The critical appraisal and risk-of-bias grading (Section 3.9; Supplementary Tables S2 and S3) bear directly on how these findings should be weighed. Selection was the dominant weakness: only one of the 12 case series [24] reported consecutive and complete inclusion, so the profiled tumors in the others are convenience samples whose relationship to the underlying patient population is unknown. This matters most for the prognostic claims: the associations of SERPINE1+ fibroblasts [21], POSTN+ myofibroblastic CAFs [22], and SFRP4-high myeloid cells [39] with poor outcome, and of APOE-negative tumor cells with lymph node metastasis [29], all arise from selected cohorts in which the direction and magnitude of selection cannot be assessed, and in three of them [21,22,39] the survival data come from external bulk cohorts or public datasets rather than from the profiled patients. Eight case series reported no outcome or follow-up for the profiled patients [20,21,22,29,31,32,35,38], four reported no demographics [20,27,35,38], and nine compared groups at the level of spots or cells rather than patients, or did not state the unit of analysis [20,27,28,29,31,32,35,38,39]; confounding by age, sex, tumor size, or stage therefore cannot be judged, the reported *p* values overstate the evidence for between-group differences, and “progression” or “aggressive” in these reports denotes a histologic rather than a clinical endpoint. The two reanalyses [26,30] share one source dataset [21] and reported neither platform-level quality control nor code, so their signatures are computational hypotheses from a single cohort. Three of the four analytical cross-sectional studies [23,33,34], the source of the MAP score, the proteogenomic DTC subtypes, and the sex-specific immune findings, were rated low concern, but their spatial components were small, undescribed subsets (eight, four, and nine patients or sections), and only Xu et al. [23] addressed confounding. The one finding replicated beyond two cohorts, tumor-microenvironment remodeling with dedifferentiation, drew support from one study graded at low risk of bias [24] and four at high risk [20,21,25,39] and from three designs; its consistency across cohorts and designs makes an artifact of any single cohort unlikely, but the five cohorts together comprise only 41 spatially profiled patients. No study was excluded on these grounds; as stated in the Methods, these judgments grade reporting completeness and structural sources of bias, not the validity of each study’s molecular observations.

The deficiencies identified in Section 3.9 define the priorities for future studies: consecutive enrollment from a defined surgical population, demographics and follow-up for every profiled patient, standardized tissue processing and analytic pipelines, orthogonal validation, and clinically meaningful endpoints (recurrence, metastasis, treatment response, survival), with emphasis on PDTC, ATC, medullary carcinoma, and distant metastases. Pairing whole-transcriptome discovery with imaging-based single-cell validation is the most direct route to reproducible spatial biomarkers.

## 5. Conclusions

Spatial transcriptomics is an emerging and powerful approach for studying thyroid cancer biology in its native tissue context. Across the 21 included studies, spatial methods revealed organized programs of tumor-microenvironment remodeling, dedifferentiation, immune evasion, fibroblast activation, invasion, metastasis, and ATC-associated biomarker expression. Current evidence remains limited by small cohorts, concentration on a limited number of platforms, heterogeneous analytic methods, and incomplete clinical outcome data. Nevertheless, spatial transcriptomics provides a promising framework for linking molecular states to histologic architecture and may ultimately improve thyroid cancer classification, prognostication, and therapeutic targeting.

Future research should move beyond spatial discovery toward biological validation and clinical translation. Integration of spatial transcriptomics with genomic, proteomic, histopathologic, and clinical data may ultimately enable spatially informed biomarkers for patient stratification and precision therapy. Larger, standardized studies with rigorous validation and longitudinal clinical annotation will be essential to translate these spatial discoveries into clinically actionable strategies.

## Figures and Tables

**Table 1:** Characteristics and principal findings of the 21 included spatial-transcriptomics studies in thyroid cancer.

Study (Year) [Ref.]	Platform	Subtype(s)	Patients/Specimens (*n*)	Key Spatial Finding
Haq et al. 2025 [19]	GeoMx DSP (NanoString) + TMA IHC	ATC (vs. PTC/non-anaplastic)	1 index case (ATC coexisting with PTC) for ST; IHC validation on TMAs across tumor types	Eight DEG mRNA + protein markers for ATC progression/dedifferentiation (e.g., COL7A1, LAMC2, SPHK1, SRPX2); distinguish ATC from follicular-cell-derived carcinomas (ST single case; protein-level confirmation in a large cohort)
Ji et al. 2026 [20]	10x Visium + scRNA-seq + functional assays	PTC → PDTC/ATC	3 surgical specimens (14,191 spots); in vitro/in vivo functional validation	CAFs in poorly-differentiated regions upregulate glycolysis; ZFP57–PKM2 lactate axis promotes dedifferentiation (functionally tested); resveratrol reverses it
Liao et al. 2025 [21]	10x Visium + scRNA-seq + mIF	PT, PTC, LPTC, ATC	13 patients; 17 ST sections (4 PT, 5 PTC, 4 LPTC, 4 ATC); scRNA-seq on 9 samples; mIF validation; in-house cohort (*n* = 50) and TCGA	TME remodeling across progression; 3 thyrocyte meta-clusters (TG+IYG+, HLA-DR+, APOE+APOC1+); SERPINE1+ fibroblasts associated with malignant progression/prognosis; stage-specific leading-edge remodeling; SERPINE1–PLAUR fibroblast–macrophage interaction inferred in ATC
Loberg et al. 2026 [22]	Visium (FFPE) + scRNA-seq atlas + mIF/IHC + bulk RNA-seq cohorts	WDTC, ATC, composite WDTC/ATC, pediatric DSV	28 ST sections from 26 tumors (13 PTC: 8 pediatric RET-fusion and 5 adult BRAF V600E; 15 ATC, 7 composite WDTC/ATC); 8 ATC sections shared with Xu et al. [23]; scRNA-seq atlas of 81 published samples; mIF/IHC; 5 bulk RNA-seq cohorts	Defines POSTN+ myofibroblast CAFs intimately associated with invasive tumor cells; associated with poor prognosis, lymph-node metastasis, and BRAF V600E; prognostic fibroblast subpopulations (myCAF signature prognostic in 5 bulk cohorts); inflammatory CAFs distant from tumor
Ning et al. 2025 [24]	Visium (spRNA-seq) + WES + IHC + functional assays	DTC → PDTC → ATC	7 samples with coexisting ATC/PDTC/DTC regions; WES; IHC; in vitro mechanistic experiments	ATC regions show immune suppression, angiogenesis, ECM remodeling; adjacent DTC shares ATC-level mutational burden → early genomic priming/clonal dedifferentiation continuum; TYMP+ TAMs enriched in ATC regions; PDCD4 loss → eIF4A-dependent immunosuppressive program (functionally tested)
Seok et al. 2025 [25]	Visium CytAssist (FFPE) + IHC	PTC, FTC, PDTC, ATC	12 patients (2 PTC, 2 FTC, 5 PDTC, 3 ATC); 1 section per patient; IHC validation	TME remodeling across differentiation states; CellDART deconvolution; myeloid cells and CAFs increase and NK/endothelial cells decrease with dedifferentiation; MDSC score inversely related to TDS; PROGENy/REACTOME pathway shifts (JAK-STAT, VEGF) with dedifferentiation
Su et al. 2026 [26]	Public ST (GEO) + scRNA reanalysis	Thyroid cancer	Reanalysis: ST from GSE250521 (Liao et al. [21]); scRNA-seq from GSE60542; TCGA-THCA; no new patients	APOE/NCF1-associated immunosuppressive niche and its prognostic signature (computationally inferred; overlapping dataset)
Wang et al. 2024 [27]	Visium + scRNA-seq + IHC/mIHC	PTC (progressive vs. non-progressive)	14 patients/18 specimens (scRNA-seq); ST on 2 patients (1 non-progressive, 1 progressive PTC); IHC in 101 additional PTCs and mIHC in 10; independent bulk dataset (*n* = 502); same patient cohort as [28]	LAMP3+ dendritic cell–T-cell subpopulation interactions associated with immune evasion in progressive PTC
Xiao et al. 2025 [29]	10x Visium + scRNA-seq + IHC + functional assays	PTC (advanced, LN metastasis)	8 patients (7 PTC, 1 MTC); 14 scRNA-seq samples; 8 ST samples from 4 patients (those with LNM; selection not described); in vitro/in vivo assays	APOE(–) tumor-cell subpopulation associated with pathological lymph-node metastasis; APOE modulates proliferation/invasion (functionally tested)
Jiang et al. 2026 [30]	Public ST (GSE250521) + scRNA + bulk (TCGA, GSE60542)	PTC (lymph-node metastasis)	Reanalysis: ST from GSE250521 (Liao et al. [21]); no new patients	LNM signature; random-forest model for preoperative N1/N0 stratification; disseminated clones escape immunity via antigen-presentation downregulation (computationally inferred; overlapping dataset)
Xu et al. 2023 [23]	Visium + DNA/RNA-seq + multiplex IF	Aggressive thyroid malignancy	251 patients/312 samples (sequencing); ST on 8 ATC sections and mIF on 33 ATC blocks (selection not described)	Molecular Aggression and Prediction (MAP) score integrating the immune microenvironment improves prognostication over high-risk mutations alone
Zhang et al. 2025 [28]	10x Visium + scRNA-seq + mIF + functional assays	PTC/PTMC	19 samples (4 para-tumor, 4 non-progressive PTMC, 5 progressive PTMC, 6 progressive PTC); from 15 patients; ST on 6 patients (2 non-progressive PTMC, 2 progressive PTMC, 2 progressive PTC); in vitro/in vivo validation; same patient cohort as [27]	PROS1–MERTK axis promotes PTMC progression through tumor-microenvironment crosstalk (functionally tested)
Zheng et al. 2025 [31]	Visium (SRT) + scRNA-seq	PTC	2 patients (1 with, 1 without LNM); 4 ST sections (tumor + para-tumor per patient); scRNA-seq of 6 samples	Resolves spatial evolution/heterogeneity of PTC underlying malignancy and metastasis; improves molecular classification; enhanced aerobic metabolism and suppressed translation programs (exploratory, 2 patients)
Yan et al. 2024 [32]	10x Visium	PTC	5 patients (Section 2.1 of article Methods section); 5 ST sections	Prognosis-associated cellular heterogeneity; spatial RNA-clinical signature genes map cell-type distributions across sections; tumor focus No. 2 and ligand–receptor interactions associated with relapse-free survival
Zhang et al. 2026 [33]	Proteogenomics + spatial transcriptomics + scRNA-seq	Advanced DTC	113 patients (proteogenomic discovery cohort); scRNA-seq in an independent validation cohort of 10 patients, 4 of whom were also profiled by ST (2 CC1, 1 CC2, 1 CC3; selection not described)	Three DTC subtypes (canonical, stromal, immunogenic); ML classifier; ST maps thyroid-differentiation, immune and stromal scores
Shobab et al. 2026 [34]	GeoMx DSP (NanoString) + flow cytometry	Differentiated TC	27 patients (prospective; 16 women/11 men); GeoMx in 9 patients (81 ROIs: normal, tumor border, tumor core)	Sex-specific immune TME; males show more dividing NK and TIGIT+CD8 T cells (flow cytometry) and reduced HLA-DRB expression (GeoMx); sex differences in immune-gene expression
Wang et al. 2026 [35]	10x Visium + scRNA-seq + functional assays	Thyroid cancer (THCA); in situ vs. metastatic	Number of ST patients and sections not stated (scRNA-seq: 2 in-gland and 2 distant-metastatic tumors; bulk RNA-seq: 7 patients); in vitro co-culture; murine xenograft and lung-metastasis models	CXCL8+ monocyte/SDC1+ tumor-stem-cell interaction network associated with remote metastatic transfer; JAK-STAT activation (functionally tested in models)
Condello et al. 2024 [36]	10x Visium (CytAssist)	FTC	1 patient (single case)	Clone-specific dysregulation of extracellular-matrix genes at the invasive (capsular/vascular) front vs. indolent core (hypothesis-generating)
Suzuki et al. 2024 [37]	Visium CytAssist (FFPE) + IHC	FTC (minimally invasive)	1 patient (ST); CD74 IHC in 36 FTCs	CD74-high subpopulation at the invasive area; CD74 IHC higher in invasive/peripheral than central tumor regions; proposed candidate marker of invasion (follicular adenomas not examined)
Li et al. 2026 [38]	Spatial metabolomics (AFADESI-MSI) + Visium	PTC + lymph-node metastasis	5 patients with or without LNM (Results; the Methods describe 3 + 3); TCGA validation; zebrafish xenograft models	Integrated spatial metabolic–transcriptional map of PTC tumorigenesis and LN metastasis; dysregulated arginine–polyamine, glycolytic and lipid metabolic pathways (functionally tested in zebrafish)
Wang et al. 2025 [39]	Visium + immunofluorescence	PTC, ATC, ATC-LM, ATC-GM (+ nodular goiter)	8 FFPE sections from 6 patients (3 from one index patient with ATC-PTC, nodal and gastric metastases; 2 nodular goiter; 2 PTC; 1 ATC); IF validation in a second cohort (11 PTC, 8 ATC, 6 gastric cancers with paired nodal metastases)	SFRP4-high tumor-specific myeloid cells associated with dedifferentiation and poor prognosis; localize near CD44+ tissue stem cells; CD44+PKHD1L1+ cells enriched in ATC metastases

Studies are summarized according to the spatial profiling platform used, thyroid cancer subtype evaluated, and principal spatial or tumor microenvironment finding. Patients/ Specimens (*n*): number of unique patients (biological replicates) and number of tissue sections, capture areas, or regions of interest profiled by spatial transcriptomics; Abbreviations: ATC, anaplastic thyroid carcinoma; DTC, differentiated thyroid carcinoma; FTC, follicular thyroid carcinoma; PDTC, poorly differentiated thyroid carcinoma; PTC, papillary thyroid carcinoma; scRNA-seq, single-cell RNA sequencing; ST, spatial transcriptomics; TME, tumor microenvironment.

**Table 2:** Strength of evidence for the principal spatial-transcriptomic findings in thyroid cancer.

Finding	Subtype(s)	Studies	Indep. Cohorts (*n*)	Orthogonal/Functional Validation	Indep. Clinical Validation	Evidence Category
TME remodeling (immune suppression, angiogenesis, ECM) accompanying dedifferentiation	PTC, LPTC, FTC, PDTC, ATC (multi-subtype)	[20,21,24,25,39]	5	mIF [21]; IHC [24,25]; IF [39]; functional: yes [20,24]	None	Replicated across independent cohorts with orthogonal validation; functional support in two studies
Activated CAF programs at invasive/dedifferentiated regions (SERPINE1+; POSTN+; glycolytic ZFP57–PKM2)	PTC/LPTC/ATC [21]; WDTC/ATC/pediatric DSV [22]; PTC→PDTC/ATC [20] (multi-subtype)	[20,21,22]	3 (each marker program: 1)	mIF [21]; mIF/IHC [22]; functional: yes [20]	None	CAF activation replicated with orthogonal validation; each marker program single-cohort; SERPINE1+/POSTN+ prognostic association only
APOE-linked tumor/immunosuppressive states	PTC, LPTC, ATC [21]; PTC with LNM [29]; TC [26]	[21,26,29]	2 ([26] reanalyzes [21])	mIF [21]; IHC [29]; functional: yes [29]	None	Replicated in two cohorts with opposite directionality; one functional study
Organized immune-evasion niches/DC–T cell interactions	PTC [27]; dataset of [21] via [26,30]	[26,27,30]	2 ([26,30] reanalyze [21])	IHC/mIHC [27]; functional: no	None	Associative; expression-inferred interactions with protein-level confirmation in one cohort
Invasive-front ECM dysregulation in follicular tumors	FTC [36]; FTC [37] (subtype-specific)	[36,37]	2	CD74 IHC in 36 additional FTCs [37]; functional: no	None	Replicated in two cohorts; IHC extension in one; associative
LNM-associated tumor, immune, and metabolic programs	PTC (subtype-specific)	[29,30,38]	2 ([30] reanalyzes [21])	IHC [29]; functional: yes [29,38]	None ([30,38]: TCGA validation)	Heterogeneous programs; one functional study
Tumor-specific SFRP4-high myeloid cells in dedifferentiated/metastatic disease	PTC, ATC, ATC metastases	[39]	1	IF [39]; functional: no	None	Single cohort; orthogonal IF
Ligand–receptor axes (PROS1–MERTK; CXCL8–SDC1)	PTMC [28]; TC [35] (subtype-specific)	[28,35]	1 each	mIF [28]; functional: yes [28,35] (in vitro; murine models)	None	Single-cohort each; functionally supported by the originating group; not independently replicated
Sex-specific immune TME	DTC (subtype-specific)	[34]	1	Flow cytometry [34]; functional: no	None	Single cohort; orthogonal validation
Prognostic/classification scores (MAP score; proteogenomic DTC subtypes; spatial RNA-clinical signature)	Aggressive TC [23]; advanced DTC [33]; PTC [32]	[23,32,33]	1 each	mIF [23]; own-group independent ST/scRNA-seq cohort [33]; functional: no	None by independent investigators	Single-cohort; not externally validated
ATC digital-spatial-profiling mRNA/protein markers	ATC vs. PTC/non-anaplastic (subtype-specific)	[19]	1	Protein DSP, same specimens; IHC on TMAs across tumor types [19]; functional: no	None	Single cohort; within-study protein concordance

Independent cohorts: number of distinct patient cohorts supporting the finding; reanalyses of a dataset generated by an included primary study (GSE250521, Liao et al. [21]) are not counted as independent; Loberg et al. [22] and Xu et al. [23] share eight anaplastic-carcinoma Visium sections, and Wang et al. 2024 [27] and Zhang et al. 2025 [28] report the same patient cohort, so each pair is counted once where both appear. Orthogonal validation: protein-level or cytometric confirmation of the spatial finding within the study (IHC, IF, protein DSP, flow cytometry); functional: whether. Abbreviations: ATC, anaplastic thyroid carcinoma; CAF, cancer-associated fibroblast; DC, dendritic cell; DSP, digital spatial profiling; DSV, diffuse sclerosing variant; DTC, differentiated thyroid carcinoma; ECM, extracellular matrix; FTA, follicular thyroid adenoma; FTC, follicular thyroid carcinoma; IF, immunofluorescence; IHC, immunohistochemistry; LNM, lymph node metastasis; LPTC, locally advanced papillary thyroid carcinoma; MAP, Molecular Aggression and Prediction; PDTC, poorly differentiated thyroid carcinoma; PTC, papillary thyroid carcinoma; PTMC, papillary thyroid microcarcinoma; TC, thyroid cancer; TME, tumor microenvironment; WDTC, well-differentiated thyroid carcinoma.

**Table 3:** Principal spatial transcriptomics platforms and their reported use in thyroid cancer.

Platform	Class	Resolution	Coverage	FFPE	Sources	Use in Thyroid Cancer
Visium (10x)	Capture	55 µm spots	Transcriptome-wide	Yes	[7,14]	Most-used platform; TME, progression, and invasion
Visium CytAssist	Capture	55 µm spots	Transcriptome-wide	Yes	[22,25,36,37]	FFPE differentiation-state and invasive-front mapping
Slide-seqV2	Capture	~10 µm beads	Transcriptome-wide	No	[52]	Not yet reported
Stereo-seq	Capture	Submicron bins	Transcriptome-wide	Limited	[53]	Not yet reported
Seq-Scope	Capture	Submicron pixels	Transcriptome-wide	Limited	[54]	Not yet reported
Xenium (10x)	Imaging	Subcellular	Targeted panel	Yes	[55]	Emerging validation platform
CosMx (NanoString)	Imaging	Subcellular	Targeted RNA/protein panels	Yes	[17]	Not yet reported in included studies
MERFISH/seqFISH+	Imaging	Subcellular	Hundreds to thousands of targets	Limited	[15,16]	Not yet reported in included studies
GeoMx DSP (NanoString)	Region-based	Histology-defined ROI	Whole transcriptome or targeted panels; protein	Yes	[18]	Immune profiling and ATC biomarker discovery

FFPE, formalin-fixed paraffin-embedded; ROI, region of interest; TME, tumor microenvironment. Resolution and coverage are representative and may vary by assay configuration. ‘Use in thyroid cancer’ refers to the thyroid cancer studies included in this review.

## Data Availability

No new primary data were generated. All data supporting this review are derived from the included published studies and are presented within the article and its Supplementary Materials; the full search strategies, per-study characteristics, and item-level critical-appraisal ratings and risk-of-bias judgments are provided in Supplementary Tables S1–S4.

## Supplementary Materials

The following supporting information can be downloaded at: https://www.mdpi.com/article/10.3390/jcm15197636/s1. Table S1: Database search strategies; Table S2: Item-level critical appraisal of the 21 included studies (panel A, JBI checklist for case series; panel B, revised JBI tool for analytical cross-sectional studies; panel C, JBI checklist for case reports; panel D, seven-item framework for reanalyses of publicly available data); Table S3: Domain-level risk-of-bias grading of the 21 included studies; Table S4: PRISMA 2020 checklist. References [19,20,21,22,23,24,25,26,27,30,31,32,41,42,43,44,45,46,47,48,49,50,51] are cited in the Supplementary Materials.

## Abbreviations

The following abbreviations are used in this manuscript:

AFADESI-MSI	Air flow-assisted desorption electrospray ionization mass spectrometry imaging
APOC1	Apolipoprotein C1
APOE	Apolipoprotein E
ATC	Anaplastic thyroid carcinoma
ATC-GM	Anaplastic thyroid carcinoma gastric metastasis
ATC-LM	Anaplastic thyroid carcinoma lymph node metastasis
BRAF	B-Raf proto-oncogene, serine/threonine kinase
CAF	Cancer-associated fibroblast
CCL18	C-C motif chemokine ligand 18
CD4	Cluster of differentiation 4
CD44	Cluster of differentiation 44
CD74	Cluster of differentiation 74
CD8	Cluster of differentiation 8
COL7A1	Collagen type VII alpha 1 chain
CSV	Comma-separated values
CXCL8	C-X-C motif chemokine ligand 8 (interleukin-8)
DC	Dendritic cell
DEG	Differentially expressed gene
DNA	Deoxyribonucleic acid
DOI	Digital object identifier
DPYSL3	Dihydropyrimidinase-like 3
DSP	Digital spatial profiling
DSV	Diffuse sclerosing variant
DTC	Differentiated thyroid carcinoma
ECM	Extracellular matrix
eIF4A	Eukaryotic initiation factor 4A
EMT	Epithelial–mesenchymal transition
FAIR	Findable, Accessible, Interoperable, and Reusable
FAP	Fibroblast activation protein
FFPE	Formalin-fixed paraffin-embedded
FTA	Follicular thyroid adenoma
FTC	Follicular thyroid carcinoma
GEO	Gene Expression Omnibus
GRADE	Grading of Recommendations, Assessment, Development, and Evaluations
HLA-DR	Human leukocyte antigen-DR
HLA-DRB	Human leukocyte antigen-DR beta chain
IF	Immunofluorescence
IHC	Immunohistochemistry
ITIM	Immunoreceptor tyrosine-based inhibitory motif
JAK-STAT	Janus kinase–signal transducer and activator of transcription
JBI	Joanna Briggs Institute
LAMB3	Laminin subunit beta 3
LAMC2	Laminin subunit gamma 2
LAMP3	Lysosome-associated membrane protein 3
LAMP5	Lysosome-associated membrane protein family member 5
LN	Lymph node
LNM	Lymph node metastasis
LPTC	Locally advanced papillary thyroid carcinoma
MAP	Molecular Aggression and Prediction
MDSC	Myeloid-derived suppressor cell
MERFISH	Multiplexed error-robust fluorescence in situ hybridization
MERTK	MER tyrosine kinase
mIF	Multiplex immunofluorescence
mIHC	Multiplex immunohistochemistry
ML	Machine learning
MRC1	Mannose receptor C-type 1
mRNA	Messenger RNA
MTC	Medullary thyroid carcinoma
myCAF	Myofibroblastic-cancer-associated fibroblast
NCF1	Neutrophil cytosolic factor 1
NK	Natural killer
NR	Not reported
NTRK	Neurotrophic receptor tyrosine kinase
PDCD4	Programmed cell death 4
PDTC	Poorly differentiated thyroid carcinoma
PKHD1L1	Polycystic kidney and hepatic disease 1-like 1
PKM2	Pyruvate kinase M2
PLAUR	Plasminogen activator, urokinase receptor
POSTN	Periostin
PRISMA	Preferred Reporting Items for Systematic Reviews and Meta-Analyses
PROS1	Protein S
PT	Para-tumor thyroid tissue
PTC	Papillary thyroid carcinoma
PTMC	Papillary thyroid microcarcinoma
RAS	Rat sarcoma family of small GTPases
RET	RET proto-oncogene
RIS	Research Information Systems file format
RNA	Ribonucleic acid
RNA-seq	RNA sequencing
ROI	Region of interest
scRNA	Single-cell RNA
scRNA-seq	Single-cell RNA sequencing
SDC1	Syndecan-1 (CD138)
seqFISH	Sequential fluorescence in situ hybridization
SERPINE1	Serpin family E member 1 (plasminogen activator inhibitor-1)
SFRP4	Secreted frizzled-related protein 4
SPHK1	Sphingosine kinase 1
spRNA-seq	Spatial RNA sequencing
SRT	Spatially resolved transcriptomics
ST	Spatial transcriptomics
TBSRTC	The Bethesda System for Reporting Thyroid Cytopathology
TC	Thyroid cancer
TCGA	The Cancer Genome Atlas
TDS	Thyroid differentiation score
TERT	Telomerase reverse transcriptase
TG	Thyroglobulin
THCA	Thyroid carcinoma
TIGIT	T-cell immunoreceptor with immunoglobulin and ITIM domains
TMA	Tissue microarray
TME	Tumor microenvironment
TP53	Tumor protein p53
TYMP	Thymidine phosphorylase
VEGF	Vascular endothelial growth factor
WDTC	Well-differentiated thyroid carcinoma
WES	Whole-exome sequencing
WHO	World Health Organization
ZFP57	Zinc finger protein 57
