# Supplementary material for: Spatial Transcriptomics in Thyroid Cancer: A PRISMA-Style Systematic Review of Platforms, Architecture, and Clinical Applications
Source: J Clin Med. 2026 Oct 2;15(19):7636. doi: 10.3390/jcm15197636 (PMC13666090; doi:10.3390/jcm15197636)
Supplement: Supplementary file 1 [file jcm-15-07636-s001.zip › jcm-4503198-supplementary.pdf]

Table S1. Database Search Strategies.

Database (Interface)	Search strategy
**PubMed / MEDLINE (NCBI)**	(<br>"spatial transcriptomic*"[tiab] OR "spatially resolved transcriptom*"[tiab]<br>OR "spatial gene expression"[tiab] OR "spatial transcriptome"[tiab]<br>OR "spatial omics"[tiab] OR "spatially resolved RNA"[tiab]<br>OR "in situ sequencing"[tiab] OR "digital spatial profiling"[tiab]<br>OR "spatial molecular imaging"[tiab]<br>OR Visium[tiab] OR "Slide-seq"[tiab] OR "Stereo-seq"[tiab] OR "Seq-Scope"[tiab]<br>OR Xenium[tiab] OR CosMx[tiab] OR MERFISH[tiab] OR seqFISH[tiab] OR GeoMx[tiab]<br>)<br>AND<br>(<br>"Thyroid Neoplasms"[Mesh]<br>OR "thyroid cancer*"[tiab] OR "thyroid carcinoma*"[tiab] OR "thyroid neoplasm*"[tiab]<br>OR "thyroid tumo*"[tiab] OR "thyroid malignan*"[tiab]<br>OR (thyroid[tiab] AND papillary[tiab]) OR (thyroid[tiab] AND follicular[tiab])<br>OR (thyroid[tiab] AND medullary[tiab]) OR (thyroid[tiab] AND anaplastic[tiab])<br>)<br>AND English[lang]
**Scopus (Elsevier)**	(<br>TITLE-ABS-KEY ("spatial transcriptomic*" OR "spatially resolved transcriptom*"<br>OR "spatial gene expression" OR "spatial transcriptome" OR "spatial omics"<br>OR "in situ sequencing" OR "digital spatial profiling" OR "spatial molecular imaging"<br>OR "Visium" OR "Slide-seq" OR "Stereo-seq" OR "Seq-Scope"<br>OR "Xenium" OR "CosMx" OR "MERFISH" OR "seqFISH" OR "GeoMx")<br>)<br>AND<br>(<br>TITLE-ABS-KEY ("thyroid cancer*" OR "thyroid carcinoma*" OR "thyroid neoplasm*"<br>OR "thyroid tumo*" OR "thyroid malignan*"<br>OR (thyroid W/3 papillary) OR (thyroid W/3 follicular)<br>OR (thyroid W/3 medullary) OR (thyroid W/3 anaplastic))<br>)<br>AND (LIMIT-TO (LANGUAGE , "English"))
**Web of Science Core Collection (Clarivate)**	TS=(<br>"spatial transcriptomic*" OR "spatially resolved transcriptom*"<br>OR "spatial gene expression" OR "spatial transcriptome" OR "spatial omics"<br>OR "in situ sequencing" OR "digital spatial profiling" OR "spatial molecular imaging"<br>OR "Visium" OR "Slide-seq" OR "Stereo-seq" OR "Seq-Scope"<br>OR "Xenium" OR "CosMx" OR "MERFISH" OR "seqFISH" OR "GeoMx"<br>)<br>AND TS=(<br>"thyroid cancer*" OR "thyroid carcinoma*" OR "thyroid neoplasm*"<br>OR "thyroid tumo*" OR "thyroid malignan*"<br>OR (thyroid NEAR/3 papillary) OR (thyroid NEAR/3 follicular)<br>OR (thyroid NEAR/3 medullary) OR (thyroid NEAR/3 anaplastic)<br>)<br>AND LA=(English)

All databases were searched on July 10, 2026, with no date restriction and limited to English-language records. Field tags and operators: [tiab] = title/abstract; [Mesh] = Medical Subject Headings; [lang]/LANGUAGE/LA = language limit;

*TITLE-ABS-KEY and TS = title, abstract, and keywords; W/3 (Scopus) and NEAR/3 (Web of Science) = terms occurring within three words of each other; * = truncation. After removal of 57 duplicates, 47 unique records remained for screening (see Figure 1, PRISMA flow diagram).*

Table S2. Item-level critical appraisal of the 21 included studies (panel A, JBI checklist for case series; panel B, revised JBI tool for analytical cross-sectional studies; panel C, JBI checklist for case reports; panel D, seven-item framework for reanalyses of publicly available data).

(A) JBI critical-appraisal checklist for case series (12 studies)

Study (Year) [Ref.]	1	2	3	4	5	6	7	8	9	10	Met / applicable (%)	Overall concern
Ji et al. 2026 [20]	U	Y	Y	U	U	N	U	N	Y	U	3/10 (30%)	High concern
Liao et al. 2025 [21]	U	Y	Y	U	U	Y	Y	N	U	Y	5/10 (50%)	Moderate concern
Loberg et al. 2026 [22]	N	U	Y	U	U	U	U	N	U	Y	2/10 (20%)	High concern
Ning et al. 2025 [23]	Y	Y	Y	Y	Y	Y	Y	Y	Y	Y	10/10 (100%)	Low concern
Wang et al. 2024 [26]	U	Y	Y	U	U	N	Y	Y	U	U	4/10 (40%)	High concern
Xiao et al. 2025 [27]	N	U	Y	U	U	Y	Y	N	U	U	3/10 (30%)	High concern
Zhang et al. 2025 [42]	U	Y	Y	U	U	Y	Y	Y	U	U	5/10 (50%)	Moderate concern
Zheng et al. 2025 [43]	Y	Y	Y	U	U	Y	Y	N	U	U	5/10 (50%)	Moderate concern
Yan et al. 2024 [44]	N	U	Y	U	U	Y	Y	N	U	U	3/10 (30%)	High concern
Wang et al. 2026 [47]	U	U	U	U	U	N	U	N	Y	U	1/10 (10%)	High concern
Li et al. 2026 [50]	U	Y	Y	U	U	N	U	N	U	U	2/10 (20%)	High concern
Wang et al. 2025 [51]	U	Y	Y	U	U	Y	Y	U	Y	U	5/10 (50%)	Moderate concern

(B) JBI critical-appraisal checklist for analytical cross-sectional studies (4 studies)

Study (Year) [Ref.]	1	2	3	4	5	6	7	8	Met / applicable (%)	Overall concern
Seok et al. 2025 [24]	N	Y	U	Y	U	N	N	Y	3/8 (38%)	High concern
Xu et al. 2023 [41]	Y	Y	Y	Y	Y	Y	Y	Y	8/8 (100%)	Low concern
Zhang et al. 2026 [45]	Y	Y	Y	Y	Y	U	Y	Y	7/8 (88%)	Low concern
Shobab et al. 2026 [46]	Y	Y	Y	Y	Y	U	U	Y	6/8 (75%)	Low concern

(C) JBI critical-appraisal checklist for case reports (3 studies)

Study (Year) [Ref.]	1	2	3	4	5	6	7	8	Met / applicable (%)	Overall concern
Haq et al. 2025 [19]	Y	N	U	Y	Y	Y	N	Y	5/8 (62%)	Moderate concern
Condello et al. 2024 [48]	Y	N	U	Y	U	N	N	Y	3/8 (38%)	High concern
Suzuki et al. 2024 [49]	N	N	N	U	U	N	N	Y	1/8 (12%)	High concern

(D) Seven-item framework for reanalyses of publicly available data (2 studies)

Study (Year) [Ref.]	1	2	3	4	5	6	7	Met / applicable (%)	Overall concern
Su et al. 2026 [25]	U	N	Y	Y	Y	U	Y	4/7 (57%)	Moderate concern
Jiang et al. 2026 [32]	Y	N	Y	Y	Y	U	Y	5/7 (71%)	Moderate concern

Column numbers correspond to checklist items. Ratings: Y, met; N, not met; U, unclear/not reported; NA, not applicable. Only Y counted as met. Overall concern was low (≥75% of applicable items met), moderate (50–74%), or high (<50%). Appraisal was performed by one reviewer (H.C.S.), independently checked by a second (C.S.G.), with disagreements resolved by consensus. (A) JBI case-series checklist [30]: 1, inclusion criteria; 2, reliable condition measurement; 3, valid condition identification; 4, consecutive inclusion; 5, complete inclusion; 6, demographics; 7, clinical information; 8, outcomes/follow-up; 9, site information; 10, statistical analysis. (B) JBI analytical cross-sectional checklist [31]: 1, inclusion criteria; 2, condition measurement; 3, exposure measurement; 4, outcome measurement; 5, confounders identified; 6, confounding addressed; 7, statistical analysis; 8, subjects/setting described. (C) JBI case-report checklist [30]: 1, demographics; 2, history/timeline; 3, presentation; 4, diagnostics; 5, intervention; 6, post-intervention condition; 7, adverse events; 8, take-away lessons. (D) Seven-item spatial-transcriptomic framework: 1, data provenance; 2, quality control; 3, analytic pipeline; 4, batch-effect handling; 5, validation; 6, code/data availability; 7, parameter transparency. For spatial-transcriptomic studies, absent reporting was rated N and incomplete reporting U; undescribed methods were rated U and inadequate methods N. Outcomes/follow-up required

data from profiled patients, and cross-patient analyses were considered appropriate only at the patient or section level. Overall: 4 studies had low, 7 moderate, and 10 high concern.

Table S3. Domain-level risk-of-bias grading of the 21 included studies.

Study (Year) [Ref.]	Design / instrument	Domain-level risk-of-bias judgements	Overall risk of bias
Haq et al. 2025 [19]	JBI Case Report (8 items)	Patient description: High; Diagnostic assessment: Low; Intervention and follow-up: Moderate; Interpretation: Low	Moderate
Ji et al. 2026 [20]	JBI Case Series	Selection: High; Measurement of condition: Low; Participant reporting: High; Outcome ascertainment: High; Analysis: High	High
Liao et al. 2025 [21]	JBI Case Series	Selection: High; Measurement of condition: Low; Participant reporting: Moderate; Outcome ascertainment: High; Analysis: Low	High
Loberg et al. 2026 [22]	JBI Case Series	Selection: High; Measurement of condition: Moderate; Participant reporting: High; Outcome ascertainment: High; Analysis: Low	High
Ning et al. 2025 [23]	JBI Case Series	Selection: Low; Measurement of condition: Low; Participant reporting: Low; Outcome ascertainment: Low; Analysis: Low	Low
Seok et al. 2025 [24]	JBI analytical cross-sectional (8 items)	Selection: Moderate; Classification of the exposure: High; Measurement of the outcome: Low; Confounding: High; Statistical conclusion validity: High; Comprehensiveness of reporting: Low	High
Su et al. 2026 [25]	Seven-Item Framework	Data provenance and QC: High; Analytic transparency: Low; Validation and availability: Moderate	Moderate
Wang et al. 2024 [26]	JBI Case Series	Selection: High; Measurement of condition: Low; Participant reporting: High; Outcome ascertainment: Low; Analysis: High	High
Xiao et al. 2025 [27]	JBI Case Series	Selection: High; Measurement of condition: Moderate; Participant reporting: Moderate; Outcome ascertainment: High; Analysis: High	High
Jiang et al. 2026 [32]	Seven-Item Framework	Data provenance and QC: Moderate; Analytic transparency: Low; Validation and availability: Moderate	Moderate
Xu et al. 2023 [41]	JBI analytical cross-sectional (8 items)	Selection: Low; Classification of the exposure: Low; Measurement of the outcome: Low; Confounding: Low; Statistical conclusion validity: Low; Comprehensiveness of reporting: Low	Low
Zhang et al. 2025 [42]	JBI Case Series	Selection: High; Measurement of condition: Low; Participant reporting: Moderate; Outcome ascertainment: Low; Analysis: High	High
Zheng et al. 2025 [43]	JBI Case Series	Selection: High; Measurement of condition: Low; Participant reporting: Moderate; Outcome ascertainment: High; Analysis: High	High

Study (Year) [Ref.]	Design / instrument	Domain-level risk-of-bias judgements	Overall risk of bias
Yan et al. 2024 [44]	JBI Case Series	Selection: High; Measurement of condition: Moderate; Participant reporting: Moderate; Outcome ascertainment: High; Analysis: High	High
Zhang et al. 2026 [45]	JBI analytical cross-sectional (8 items)	Selection: Low; Classification of the exposure: Low; Measurement of the outcome: Low; Confounding: Moderate; Statistical conclusion validity: Low; Comprehensiveness of reporting: Low	Low
Shobab et al. 2026 [46]	JBI analytical cross-sectional (8 items)	Selection: Low; Classification of the exposure: Low; Measurement of the outcome: Low; Confounding: Moderate; Statistical conclusion validity: High; Comprehensiveness of reporting: Low	Moderate
Wang et al. 2026 [47]	JBI Case Series	Selection: High; Measurement of condition: High; Participant reporting: High; Outcome ascertainment: High; Analysis: High	High
Condello et al. 2024 [48]	JBI Case Report (8 items)	Patient description: High; Diagnostic assessment: Low; Intervention and follow-up: High; Interpretation: Low	High
Suzuki et al. 2024 [49]	JBI Case Report (8 items)	Patient description: High; Diagnostic assessment: High; Intervention and follow-up: High; Interpretation: Low	High
Li et al. 2026 [50]	JBI Case Series	Selection: High; Measurement of condition: Low; Participant reporting: High; Outcome ascertainment: High; Analysis: High	High
Wang et al. 2025 [51]	JBI Case Series	Selection: High; Measurement of condition: Low; Participant reporting: Low; Outcome ascertainment: High; Analysis: High	High

Domains were formed by grouping the checklist items in Supplementary Table 2 by the source of bias they address. Case series: Selection, items 1, 4, 5; Measurement of condition, items 2, 3; Participant reporting, items 6, 7, 9; Outcome ascertainment, item 8; Analysis, item 10. Analytical cross-sectional (categories and bias domains of the revised JBI tool [31]): Selection, items 1, 2; Classification of the exposure, item 3; Measurement of the outcome, item 4; Confounding, items 5, 6; Statistical conclusion validity, item 7; Comprehensiveness of reporting, item 8. Case report: Patient description, items 1–3; Diagnostic assessment, item 4; Intervention and follow-up, items 5–7; Interpretation, item 8. Reanalyses (seven-item framework): Data provenance and QC, items 1, 2; Analytic transparency, items 3, 4, 7; Validation and availability, items 5, 6. Domain judgement: Low, all applicable items met; Moderate, at least half but not all items met; High, fewer than half met (unclear items counted as not met). Overall judgement: Low, no domain High and at most one Moderate; High, two or more domains High, or one High plus two or more Moderate; Moderate, otherwise. Summary: Low, 3 studies (Xu et al. 2023; Zhang et al. 2026; Ning et al. 2025); Moderate, 4 (Haq et al. 2025; Shobab et al. 2026; Su et al. 2026; Jiang et al. 2026); High, 14. Because the checklists are adapted to descriptive spatial-omics studies and the domain grouping and cut points have not been externally validated, these judgements grade reporting completeness and the structural sources of potential bias rather than the intrinsic validity of each study's molecular observations; they were not used to weight or exclude studies. JBI, Joanna Briggs Institute; QC, quality control.

Table S4. PRISMA 2020 Checklist.

PRISMA 2020 Main Checklist

Topic	No.	Item	Location where item is reported
**TITLE**			
**Title**	1	Identify the report as a systematic review.	Title, p. 1
**ABSTRACT**			
**Abstract**	2	See the PRISMA 2020 for Abstracts checklist	
**INTRODUCTION**			
**Rationale**	3	Describe the rationale for the review in the context of existing knowledge.	Introduction, §1, pp. 4–5
**Objectives**	4	Provide an explicit statement of the objective(s) or question(s) the review addresses.	Introduction, §1, p. 5
**METHODS**			
**Eligibility criteria**	5	Specify the inclusion and exclusion criteria for the review and how studies were grouped for the syntheses.	Methods, §2, p. 6
**Information sources**	6	Specify all databases, registers, websites, organisations, reference lists and other sources searched or consulted to identify studies. Specify the date when each source was last searched or consulted.	Methods, §2, p. 5; Supplementary Table 1 (last searched July 2026, no lower date limit)
**Search strategy**	7	Present the full search strategies for all databases, registers and websites, including any filters and limits used.	Methods, §2, p. 5; Supplementary Table 1
**Selection process**	8	Specify the methods used to decide whether a study met the inclusion criteria of the review, including how many reviewers screened each record and each report retrieved, whether they worked independently, and if applicable, details of automation tools used in the process.	Methods, §2, p. 6
**Data collection process**	9	Specify the methods used to collect data from reports, including how many reviewers collected data from each report, whether they worked independently, any processes for obtaining or confirming data from study investigators, and if applicable, details of automation tools used in the process.	Methods, §2, p. 6
**Data items**	10a	List and define all outcomes for which data were sought. Specify whether all results that were compatible with each outcome domain in each study were sought (e.g. for all measures, time points, analyses), and if not, the methods used to decide which results to collect.	Methods, §2, p. 6

Topic	No.	Item	Location where item is reported
	10b	List and define all other variables for which data were sought (e.g. participant and intervention characteristics, funding sources). Describe any assumptions made about any missing or unclear information.	Methods, §2, p. 6
**Study risk of bias assessment**	11	Specify the methods used to assess risk of bias in the included studies, including details of the tool(s) used, how many reviewers assessed each study and whether they worked independently, and if applicable, details of automation tools used in the process.	Methods, §2, pp. 6–7
**Effect measures**	12	Specify for each outcome the effect measure(s) (e.g. risk ratio, mean difference) used in the synthesis or presentation of results.	Not applicable – qualitative synthesis, no pooled effect measures (Methods, §2, p. 7)
**Synthesis methods**	13a	Describe the processes used to decide which studies were eligible for each synthesis (e.g. tabulating the study intervention characteristics and comparing against the planned groups for each synthesis (item 5)).	Methods, §2, p. 7
	13b	Describe any methods required to prepare the data for presentation or synthesis, such as handling of missing summary statistics, or data conversions.	Methods, §2, p. 7
	13c	Describe any methods used to tabulate or visually display results of individual studies and syntheses.	Methods, §2, p. 7; Tables 1–2
	13d	Describe any methods used to synthesize results and provide a rationale for the choice(s). If meta-analysis was performed, describe the model(s), method(s) to identify the presence and extent of statistical heterogeneity, and software package(s) used.	Methods, §2, p. 7
	13e	Describe any methods used to explore possible causes of heterogeneity among study results (e.g. subgroup analysis, meta-regression).	Not applicable – no meta-analysis
	13f	Describe any sensitivity analyses conducted to assess robustness of the synthesized results.	Not applicable – no meta-analysis
**Reporting bias assessment**	14	Describe any methods used to assess risk of bias due to missing results in a synthesis (arising from reporting biases).	Methods, §2, p. 7 (not formally assessed – qualitative synthesis)
**Certainty assessment**	15	Describe any methods used to assess certainty (or confidence) in the body of evidence for an outcome.	Methods, §2, p. 7 (GRADE not applied; JBI appraisal used)
**RESULTS**			

Topic	No.	Item	Location where item is reported
**Study selection**	16a	Describe the results of the search and selection process, from the number of records identified in the search to the number of studies included in the review, ideally using a flow diagram.	Results, §3.1, p. 9; Figure 1, p. 10
	16b	Cite studies that might appear to meet the inclusion criteria, but which were excluded, and explain why they were excluded.	Results, §3.1, p. 9; Figure 1
**Study characteristics**	17	Cite each included study and present its characteristics.	Results, §3, p. 10; Table 1
**Risk of bias in studies**	18	Present assessments of risk of bias for each included study.	Results, §3.9, p. 17; Supplementary Tables 2 and 3
**Results of individual studies**	19	For all outcomes, present, for each study: (a) summary statistics for each group (where appropriate) and (b) an effect estimate and its precision (e.g. confidence/credible interval), ideally using structured tables or plots.	Table 1, p. 10 (effect estimates not applicable – qualitative)
**Results of syntheses**	20a	For each synthesis, briefly summarise the characteristics and risk of bias among contributing studies.	Results, §§3.2–3.9, pp. 11–17
	20b	Present results of all statistical syntheses conducted. If meta-analysis was done, present for each the summary estimate and its precision (e.g. confidence/credible interval) and measures of statistical heterogeneity. If comparing groups, describe the direction of the effect.	Results of syntheses: Not applicable – no meta-analysis
	20c	Present results of all investigations of possible causes of heterogeneity among study results.	Not applicable – no meta-analysis
	20d	Present results of all sensitivity analyses conducted to assess the robustness of the synthesized results.	Not applicable – no meta-analysis
**Reporting biases**	21	Present assessments of risk of bias due to missing results (arising from reporting biases) for each synthesis assessed.	Not applicable / not formally assessed (Methods §2, p. 7; Discussion, p. 21)
**Certainty of evidence**	22	Present assessments of certainty (or confidence) in the body of evidence for each outcome assessed.	Results §3.9, p. 17; Discussion, p. 21 (formal GRADE not applied)
**DISCUSSION**			
**Discussion**	23a	Provide a general interpretation of the results in the context of other evidence.	Discussion, §4, p. 19
	23b	Discuss any limitations of the evidence included in the review.	Discussion, §4, p. 21
	23c	Discuss any limitations of the review processes used.	Discussion, §4, p. 21

Topic	No.	Item	Location where item is reported
	23d	Discuss implications of the results for practice, policy, and future research.	Discussion, §4, pp. 20–21; Conclusions, §5, p. 22
**OTHER INFORMATION**			
**Registration and protocol**	24a	Provide registration information for the review, including register name and registration number, or state that the review was not registered.	Methods, §2, p. 5 (review not registered)
	24b	Indicate where the review protocol can be accessed, or state that a protocol was not prepared.	Methods, §2, p. 5 (no separate protocol published)
	24c	Describe and explain any amendments to information provided at registration or in the protocol.	Methods, §2, p. 5 (a priori criteria, no amendments)
**Support**	25	Describe sources of financial or non-financial support for the review, and the role of the funders or sponsors in the review.	Funding statement, p. 22 (no external funding)
**Competing interests**	26	Declare any competing interests of review authors.	Conflicts of Interest statement, p. 23
**Availability of data, code and other materials**	27	Report which of the following are publicly available and where they can be found: template data collection forms; data extracted from included studies; data used for all analyses; analytic code; any other materials used in the review.	Data Availability Statement, p. 22; Tables 1–2; Supplementary Tables 1–4

PRIMSA Abstract Checklist

Topic	No.	Item	Reported?
**TITLE**			
**Title**	1	Identify the report as a systematic review.	Yes
**BACKGROUND**			
**Objectives**	2	Provide an explicit statement of the main objective(s) or question(s) the review addresses.	Yes
**METHODS**			
**Eligibility criteria**	3	Specify the inclusion and exclusion criteria for the review.	Yes
**Information sources**	4	Specify the information sources (e.g. databases, registers) used to identify studies and the date when each was last searched.	Yes
**Risk of bias**	5	Specify the methods used to assess risk of bias in the included studies.	Yes
**Synthesis of results**	6	Specify the methods used to present and synthesize results.	Yes
**RESULTS**			

Topic	No.	Item	Reported?
**Included studies**	7	Give the total number of included studies and participants and summarise relevant characteristics of studies.	Yes
**Synthesis of results**	8	Present results for main outcomes, preferably indicating the number of included studies and participants for each. If meta-analysis was done, report the summary estimate and confidence/credible interval. If comparing groups, indicate the direction of the effect (i.e. which group is favoured).	Yes
**DISCUSSION**			
**Limitations of evidence**	9	Provide a brief summary of the limitations of the evidence included in the review (e.g. study risk of bias, inconsistency and imprecision).	Yes
**Interpretation**	10	Provide a general interpretation of the results and important implications.	Yes
**OTHER**			
**Funding**	11	Specify the primary source of funding for the review.	Yes
**Registration**	12	Provide the register name and registration number.	Yes
